# The frequency of colorectal lesions in the first-degree relatives of patients with colorectal lesions among PERSIAN Guilan Cohort Study population (PGCS)

**DOI:** 10.1186/s12876-024-03177-z

**Published:** 2024-02-26

**Authors:** Somaieh Matin, Farahnaz Joukar, Saman Maroufizadeh, Mehrnaz Asgharnezhad, Paridokht Karimian, Fariborz Mansour-Ghanaei

**Affiliations:** 1 Department of Internal Medicine, School of Medicine, Imam Khomeini Hospital, Ardabil University of Medical Sciences, Ardabil, Iran; 2 Gastrointestinal and Liver Diseases Research Center, Guilan University of Medical Sciences, Razi Hospital, Sardar-Jangle Ave, 41448-95655 Rasht, Iran; 3 Department of Biostatistics and Epidemiology, School of Health, Guilan University of Medical Sciences, Rasht, Iran; 4 Department of Pathology, School of Medicine, Guilan University of Medical Sciences, Rasht, Iran

**Keywords:** Adenomatous polyps, Colorectal lesions, First-degree relatives, Malignancy, Screening

## Abstract

**Background:**

This study aimed to investigate the frequency of colorectal lesions in the first-degree relatives of patients with colorectal lesions among the Prospective Epidemiological Research Studies in Iran (PERSIAN )Guilan Cohort Study (PGCS) population.

**Methods:**

In this cross-sectional study, 162 first-degree relatives with a history of colorectal lesions were randomly selected from 52 participants in PGCS. All subjects underwent total colonoscopy by a gastroenterologist, and a pathologist evaluated colorectal biopsies. Also, individuals’ demographic information, clinical data, and dietary habits were recorded.

**Results:**

The mean age of the participants was 56.55 ± 7.04. Of 86 colon polyps, 52 neoplastic and 34 non-neoplastic polyps were observed in 56 patients (34.6%). Individuals with age > 60 years had 3.29-fold increased odds of developing colorectal polyps (OR = 3.29, 95% CI: 1.13–9.56, *P* = 0.029). The smokers were 2.73 times more susceptible to developing colorectal polyps than non-smokers (OR = 2.73, 95% CI: 1.24–6.02, *P* = 0.013). Moreover, consumption of vegetables more than three times per day was associated with decreased OR of colorectal polyp development (OR = 0.43, CI: 0.19–0.98, *P =* 0.045).

**Conclusions:**

Considering the high prevalence of neoplastic colorectal polyps among the first-degree relatives of patients with colorectal lesions, early screening is recommended for individuals with a family history of colorectal lesions.

**Supplementary Information:**

The online version contains supplementary material available at 10.1186/s12876-024-03177-z.

## Introduction

Colorectal cancer (CRC) is the third most prevalent cancer in the world among both men and women [1, 2]. Reducing mortality associated with this particular form of cancer is feasible by detecting it at an earlier stage and promptly administering the necessary treatments. Nevertheless, approximately two-thirds of cases are diagnosed during advanced stages [3, 4]. The adenoma-carcinoma sequence is a well-acknowledged mechanism that contributes to the development of colorectal cancer. Approximately two-thirds of colorectal carcinomas are estimated to originate from adenomatous polyps [5, 6]. Adenomatous polyps have high malignancy potential and are considered an important factor in the development of colorectal cancer [7].

Genetics and epigenetics affect the susceptibility of colorectal lesions such as polyps among individuals. Epigenetic factors, including male gender, upper age, higher body mass index (BMI), smoking, alcohol consumption, low level of physical activity, and low fiber dietary consumption, play an essential role in gene expression of colorectal lesions [8–10].Evidence suggests a strong association between fiber consumption and a lower rate of CRC. Besides the essential vitamins and antioxidants in fruits and vegetables, fiber enhances digestive function and reduces complications [11]. Individuals with a family history of colon cancer or adenomatous polyp are subject to higher risks of colon cancer [12–15]. The presence of colon cancer in first-degree relatives increases the incidence rate of colorectal cancer to 1.7 times as much as that of the normal population [16]. A higher incidence rate has also been observed for colorectal cancer among the first-degree relatives of patients with adenomatous polyps [17].

In contrast to individuals with a family history of colorectal cancer, the guidelines for screening individuals with a family history of polyps are inconsistent and contradictory [18, 19]. In the United States, individuals with a family history of advanced polyps are advised to undergo early screening, similar to those with a family history of colorectal cancer [18]. In contrast, the British Society of Gastroenterology recommends screening specifically for individuals with a family history of colorectal cancer and does not prioritize those with a family history of polyps [19]. To shed light on this issue, the present study aimed to investigate the frequency of colorectal lesions among the first-degree relatives of the patients with adenomatous polyp or premalignant/ malignant colorectal lesions among PERSIAN Guilan Cohort Study (PGCS) population.

## Methods

### Study design

This cross-sectional study, between November 2021 and May 2022, was conducted on 162 first-degree relatives (Including brothers, sisters, sons, and daughters) of 52 patients in PGCS who had a history of adenomatous polyp or premalignant/ malignant colorectal lesions. These patients were individuals aged above 50 years who participated in the PGCS as a part of the PERSIAN (Prospective Epidemiological Research Studies in IRAN) cohort [20] with a sample size of 10,520 males and females in Some’e Sara County (including 39 villages and urban regions), Guilan, Iran [21]. The study has been confirmed by the ethical committee of the Guilan University of Medical Sciences, Rasht, Iran (IR.GUMS.REC.1400.409), the written or verbal (in case of being illiterate) informed consent was obtained from all participants. Based on the statistical information of Guilan province, which reported the average family size in this area to be 3.13, we randomly selected three or more subjects from each family with a patient with an adenomatous polyp.

Finally, 162 subjects entered the present study, and the study’s purpose and all steps were explained to them. Subjects with a history of colectomy for any reason, inflammatory bowel diseases (IBD), colorectal cancer, dementia, and severe psychological disorders, residence in elder care homes / retirement homes, acute physical problems, or disabilities were all excluded from the study. The demographic and clinical data and dietary habits, including relation with the patient (Son-

Daughter.

Alternatively, Sister-Bother), age, gender, marital status, education level, employment status, habitat, body mass index (BMI), smoking, use of opium, alcohol consumption, hypertension, diabetes, hyperlipidemia, constipation, anorectal complaints and dietary habits such as consumption of dairy, meat, bread, vegetables, and fruits, were recorded. All individuals underwent total colonoscopy by an expert gastroenterologist with Olympus video endoscopes (Olympus CV-190, Power INPUT 220-240 V, 50/60 Hz, 150VA, JAPAN) based on the guidelines proposed for screening colorectal cancer in the endoscopy department of Razi Educational and Medical Center, Rasht, Iran. After sectioning and staining with hematoxylin and eosin, a pathologist evaluated the colorectal biopsies under a light microscope. Also, the colonoscopy findings (type, polyps, and lesion size) and pathology results were documented.

### Statistical analysis

This study expressed continuous variables as mean ± standard deviation (S.D.) and categorical variables as number (percentage). In univariable analysis, simple logistic regression was used to examine the relationship of demographic and clinical variables with the presence of colon polyps. Then, backward logistic regression analysis was applied to identify the independent risk factors for the outcome. In this analysis, the alpha-to-remove was set at 0.1. Odds ratio (OR) and 95% confidence interval (CI) were calculated. All data analyses were done with SPSS for Windows, version 16.0 (SPSS Inc., Chicago, IL, USA), and the significance level was set at 0.05.

## Results

### Characteristics of the participants

The mean age of the participants was 56.55 ± 7.04 years. Of the participants, 53.7% were male, 76.5% were married, 24.1% were illiterate, 14.2% were farmers, 30.9% were residents in rural areas, 16.7% were overweight or obese-BMI, 27.8% were smokers, 14.8% consumed opium, 6.2% consumed alcohol, 14.2% had hypertension, 4.9% had diabetes, and 11.7% had hyperlipidemia.

### Characteristics of the colorectal polyps

In total, 86 colon polyps (52 neoplastic and 34 non-neoplastic polyps) were observed in 56 (34.6%) individuals. Moreover, the frequency of having at least one small and large colorectal polyp was 31(19.9%) and 36 (22.2%), respectively (Table 1). Frequency distribution of small colorectal polyps (< 10 mm) and large colorectal polyps (> 10 mm), were 40 (46.5%) and 46 (53.5%), respectively. The most common site of small and large polyps was rectum 17 (19.8%) and transverse section of colon 11(12.8%) (Table 2).

**Table 1:** Frequency distribution of the number and size of colorectal polyps among participants of the PERSIAN Guilan cohort study (PGCS)

		Frequency	Prevalence	
			Among all subjects (*n* = 162)	Among subjects with polyps (*n* = 56)
Having Colon Polyp				
	No	106	65.4%	-
	Yes	56	34.6%	-
Polyp Number				
	1	29	17.9%	51.8%
	2	24	14.8%	42.9%
	3	3	1.9%	5.4%
Polyp size				
	At least one small polyp	31	19.9%	55.4%
	At least one large polyp	36	22.2%	64.3%

**Table 2:** Frequency distribution of colorectal polyp location among participants of the PERSIAN Guilan cohort study (PGCS)

	Small polyp (< 10 mm)	Large polyp (> 10 mm)	Total
Rectum	17 (19.8%)	4 (4.7%)	21 (24.4%)
Sigmoid Colon	9 (10.5%)	8 (9.3%)	17 (19.8%)
Descending Colon	5 (5.8%)	8 (9.3%)	13 (15.1%)
Transverse Colon	2 (2.3%)	11 (12.8%)	13 (15.1%)
Ascending Colon	3 (3.5%)	8 (9.3%)	11 (12.8%)
Cecum	3 (3.5%)	5 (5.8%)	8 (9.3%)
Hepatic Flexure	1 (1.2%)	2 (2.3%)	3 (3.5%)
Total	40 (46.5%)	46 (53.5%)	86 (100%)

Values are presented as “n (%)” Note. The percentages are calculated based on 86 polyps.

Frequency distribution of neoplastic polyps among small and large colorectal polyps was 18 (20.9%) and 34 (39.5%), respectively. Moreover, the most prevalent neoplastic small and large colorectal polyp was tubular adenoma 14 (16.3%) and 31 (36%), respectively (Table 3).

**Table 3:** Frequency distribution of pathology findings of colorectal polyps among participants of the PERSIAN Guilan cohort study (PGCS)

		Small polyp (< 10 mm)	Large polyp (> 10 mm)	Total
Neoplastic		18 (20.9%)	34 (39.5%)	52 (60.5%)
	Tubular adenoma	14 (16.3%)	31 (36%)	45 (52.3%)
	Tubulovillous	2 (2.3%)	1 (1.2%)	3 (3.5%)
	Villus	0	1 (1.2%)	1 (1.2%)
	SSP	2 (2.3%)	1 (1.2%)	3 (3.5%)
Non-neoplastic		22 (25.6%)	12 (14%)	34 (39.6%)
	Hyperplastic	22 (25.6%)	11(12.8)	33 (38.4%)
	inflammatory	0	1 (1.2%)	1 (1.2%)
Total		40 (46.5%)	46 (53.5%)	86 (100%)

Values are presented as “n (%).” Note. The percentages are calculated based on 86 polyps

### Factors associated with presence of colorectal polyps

Univariable logistic regression analysis was undertaken to identify factor associated with the presence of colon polyps (see Table 4). Participants aged > 60 years had an almost 5-fold increased odds of having colorectal polyps compared with those aged 40–50 years (OR = 5.01, 95% CI: 1.85–13.42). Cigarette smoking increased the odds of having colorectal polyps (OR = 2.16, 95% CI: 1.31–5.41). Participants with hypertension were 2.90 times likely to have colorectal polyps than other participants (OR = 2.90, 95% CI: 1.18–7.13). Increasing the consumption of vegetables and fruits deceased the odds of having colorectal polyps (OR = 0.34, 95% CI: 0.16–0.74, and OR = 0.37, 95% CI: 0.19–0.72). Overweight or Obese-BMI participants were at increased odds for having colorectal polyps compared to normal-BMI participants (OR = 2.67, 95% CI: 0.95–7.49), although this relationship was not statistically significant (*P* = 0.062).

**Table 4:** The association between factors and the presence of colorectal polyps among participants of the PERSIAN Guilan cohort study (PGCS)

		Prevalence of Colon Polyps		Simple logistic regression		Multiple logistic regression with backward elimination	
		n / N	%	OR (95% CI)	*P*	OR (95% CI)	*P*
Relationship							
	Son-Daughter	46 / 125	36.8	1			
	Sister-Bother	10 / 37	27.0	1.57 (0.70–3.54)	0.275		
Age group							
	40–50 y	7 / 38	18.4	1		1	
	50–60 y	23 / 75	30.7	1.96 (0.75–5.09)	0.168	1.30 (0.47–3.64)	0.613
	>60 y	26 / 49	53.1	5.01 (1.85–13.52)	0.001	3.29 (1.13–9.56)	0.029
Sex							
	Male	34 / 87	39.1	1.55 (0.80–2.98)	0.195		
	Female	22 / 75	29.3	1			
Marital Status							
	Married	45 / 124	36.3	1			
	Single	4 / 9	44.4	1.40 (0.36–5.50)	0.626		
	Widowed/	7 / 29	24.1	0.56 (0.22–1.41)	0.218		
Education							
	Illiterate	18 / 39	46.2	1.76 (0.76–4.05)	0.185		
	Under diploma	19 / 65	29.2	0.85 (0.39–1.82)	0.673		
	Diploma and higher	19 / 85	32.8	1			
Occupation							
	Farmer	11 / 23	47.8	1			
	Employee	13 / 37	35.1	0.59 (0.20–1.71)	0.331		
	Others	13 / 44	29.5	0.46 (0.16–1.30)	0.142		
	Housekeeper	19 / 58	32.8	0.53 (0.20–1.42)	0.208		
Place of residency							
	Urban	39 / 112	34.8	1			
	Rural	17 / 50	34.0	0.96 (0.48–1.95)	0.919		
BMI (kg/m^2^)							
	Normal	5 / 27	18.5	1		1	
	Overweight/Obese	51 / 135	37.8	2.67 (0.95–7.49)	0.062	2.74 (0.89–8.47)	0.080
Smoking							
	No	33 / 117	28.2	1		1	
	Yes	23 / 45	51.1	2.66 (1.31–5.41)	0.007	2.73 (1.24–6.02)	0.013
Opium Consumption							
	No	47 / 138	34.1	1			
	Yes	9 / 24	37.5	1.16 (0.47–2.85)	0.744		
Alcohol Consumption							
	No	52 / 152	34.2	1			
	Yes	4 / 10	40.0	1.28 (0.35–4.75)	0.710		
Hypertension							
	No	43 / 139	30.9	1			
	Yes	13 / 23	56.5	2.90 (1.18–7.13)	0.020		
Diabetes							
	No	52 / 154	33.8	1			
	Yes	4 / 8	50.0	1.96 (0.47–8.16)	0.354		
Hyperlipidemia							
	No	47 / 143	32.9	1			
	Yes	9 / 19	47.4	1.84 (0.70–4.83)	0.217		
Constipation							
	No	40	32.5	1		1	
	Yes	16	41.0	1.44 (0.69–3.03)	0.332	2.11 (0.90–4.93)	0.086
Anorectal Compliant							
	No	40 / 127	31.5	1			
	Yes	16 / 35	45.7	1.83 (0.85–3.93)	0.120		
Dairy (daily/promise)							
	< 2	34 / 93	36.6	1			
	≥ 2	22 / 69	31.9	0.81 (0.42–1.57)	0.536		
Meat (daily/promise)							
	< 2	22 / 65	33.8	1			
	≥ 2	34 / 97	35.1	1.05 (0.54–2.04)	0.874		
Bread (daily/promise)							
	< 6	4 / 10	40.0	1			
	≥ 6	52 / 152	34.2	0.78 (0.21–2.89)	0.710		
Vegetables (daily/promise)							
	< 3	45 / 107	42.1	1		1	
	≥ 3	11 / 55	20.0	0.34 (0.16–0.74)	0.006	0.43 (0.19–0.98)	0.045
Fruits (daily/promise)							
	< 2	32 / 67	47.8	1		1	
	≥ 2	24 / 95	25.3	0.37 (0.19–0.72)	0.003	0.49 (0.23–1.06)	0.069

OR: Odds Ratio; CI: Confidence Interval

Multiple logistic regression with backward elimination (*p*-value for removal > 0.1) was undertaken to identify factors independently associated with the presence of colorectal polyps. According to this analysis, individuals with age > 60 years had 3.29-fold increased odds of developing colorectal polyps compared with younger ages (OR = 3.29, 95% CI: 1.13–9.56). The cigarette smoking was a risk factor that increased the odds of developing colorectal polyps by about 173% (OR = 2.73, 95% CI: 1.24–6.02). Consumption of vegetables more than three times a week was associated with decreased OR of colorectal polyp developing (OR = 0.43, CI: 0.19–0.98).

Moreover, results showed that being overweight/obese, having constipation and low consumption of vegetables were associated with an increased odds of having colorectal polyps, although these relationships were not statistically significant (*P* = 0.080, *P* = 0.086, and *P* = 0.069, respectively).

## Discussion

The detection of high-risk people and performing appropriate screening tests on them has important health-related and economic advantages as regards the prevention of colorectal cancer [22]. In the present study, the prevalence of colorectal polyps among participants was 34.6%. More than 60% of the detected colorectal polyps were neoplastic. The prevalence of neoplastic colon polyp among the siblings of the patients with advanced adenoma was reported to be 11.5% in the study conducted by NG SC et al. [23] and 4.4% in the study conducted by Cottet et al. [24] The higher prevalence of neoplastic polyp in the current study might be the result of different regional factors, dietary habits, genetic features of the subjects, or higher accuracy in detecting colorectal polyps. In addition, in our study, the most prevalent neoplastic colorectal polyp was tubular adenoma, which is consistent with the findings reported in previous studies [24–26]. Based on the current study’s findings, small polyps were most frequently observed in the rectum; however, large polyps had the highest frequency in the transvers section of the colon, consistent with the findings of the previous studies [27–29].

The results revealed that individuals aged 60 had a higher probability of developing colorectal polyp. This finding is consistent with the findings reported in most previous studies [24–26, 30, 31]. In the present study, the probability of developing colorectal polyps was higher in smokers than in non-smokers. In line with our findings, previous studies [32–34] have also indicated that smoking is strongly associated with a higher risk of colorectal polyps. The carcinogens present in tobacco increase the incidence of colorectal cancer [35]. Therefore, it can be argued that smoking causes an increase in the incidence of colorectal polyps and, in turn, colorectal cancer either independently or in combination with other factors, such as the family history of adenomatous polyp. The results of the current study also indicated that the odds of colorectal polyp developing were higher among people suffering from hypertension as compared to individuals without it. This finding was inconsistent with the finding reported by Ng SC et al. [23]. This observation might be due to the higher prevalence of hypertension at upper ages, which leads to a higher probability of polyp development in the affected patients.

The results of this study also revealed that the increase in the consumption of vegetables and fruits significantly reduced the likelihood of developing colorectal polyps. The association between dietary fiber and colon cancer has been under investigation for three decades; however, no definite association has been recognized [36, 37]. Aune et al. reported that a higher proportion of fiber in everyday dietary regimens can reduce the recurrence rate of colorectal adenomas [38]. Similar to the process of carcinogenesis, the possible mechanism is that the fiber undergoes anaerobic glycolysis and, therefore, can be oxidized and converted into short-chain fatty acid. The resulting fatty acid can be key in inhibiting cell proliferation and facilitating apoptosis or cell differentiation [39].

Therefore, it is expected that the dietary habits containing vegetables and fruits due to having higher fiber and containing calcium, selenium, vitamins, folic acid, carotenoids, and plant phenolic play a protective role against colorectal cancer [40]. In some studies [41, 42], an association has been observed between the consumption of red meat and the recurrence of various colorectal adenomas. In the present study, however, no similar association was observed, the reason for which might be the dietary habits of subjects in this region with a high consumption of local vegetables and fruits. The limitations of the present study include the small sample size, which may have reduced the statistical power to detect factors associated with colorectal polyps. Second, the particular conditions of the province in terms of the COVID-19 pandemic, which limited our access to more participants, and the lack of a control group were limitations of the study. It is suggested that studies be conducted in a larger population considering the amount of each food item.

## Conclusion

The age of 60 years, cigarette smoking, having diabetes, and consumption of vegetables more than three times per day were associated with an increased likelihood of colorectal polyp developing. Also, regarding the high prevalence of neoplastic colorectal polyps among the first-degree relatives of patients with adenomatous polyps or premalignant/ malignant colorectal lesions, it seems reasonable to carry out early screening for individuals with a family history of colorectal lesions. Moreover, setting up the benefits of daily dietary fiber consumption from childhood could be considered a national policy to prevent CRC risk.

### Electronic supplementary material

Below is the link to the electronic supplementary material.

Supplementary Material 1

Supplementary Material 2

## Data Availability

The datasets used and/or analyzed during the present study are available from the corresponding author on reasonable request.
