# Supplementary material for: The frequency of colorectal lesions in the first-degree relatives of patients with colorectal lesions among PERSIAN Guilan Cohort Study population (PGCS)
Source: BMC Gastroenterol. 2024 Feb 26;24:88. doi: 10.1186/s12876-024-03177-z (PMC10898130; doi:10.1186/s12876-024-03177-z)
Supplement: Supplementary file 1 — Supplementary Material 1 [file 12876_2024_3177_MOESM1_ESM.docx]

Questionnaire number:………… The date of completion of the questionnaire:……….

Address and contact number:……

**First part: Demographic characteristics**

1. Name and surname:…………..
2. Age:**……………..**
3. Gender: Male🞎 Female🞎
4. Marital Status: Married 🞎 Single 🞎 Widowed🞎
5. BMI:…………kg/m^2^
6. Occupation :Farmer 🞎 Employee 🞎 Others🞎 Housekeeper
7. Education: Illiterate🞎 Under diploma 🞎 Diploma and higher🞎
8. Place of residency: Urban🞎 Rural🞎
9. Relationship with the individual: Father🞎 Mother 🞎 Sister🞎 Brother🞎 Child🞎
10. Smoking: No🞎 Yes🞎
11. Opium Consumption: No🞎 Yes🞎
12. Alcohol Consumption: No🞎 Yes🞎
13. Preliminary Disease: Hypertension🞎 Diabetes 🞎 Hyperlipidemia 🞎 Constipation 🞎 Anorectal Compliant🞎
14. History of colonoscopy: No🞎 Yes🞎
15. Cause of colonoscopy: screening🞎 Digestive problem🞎 The type of problem that led to the colonoscopy…

**Second part: Nutritional habits**

Dairy consumption (promise/day)

< 2

≥ 2

Meat consumption (promise/day)

< 2

≥ 2

Bread consumption (promise/day)

< 6

≥ 6

Vegetables consumption (promise/day)

<

3

≥ 3

Fruits consumption (promise/day)

< 2

≥ 2

**Third part: Bristol Stool Chart**


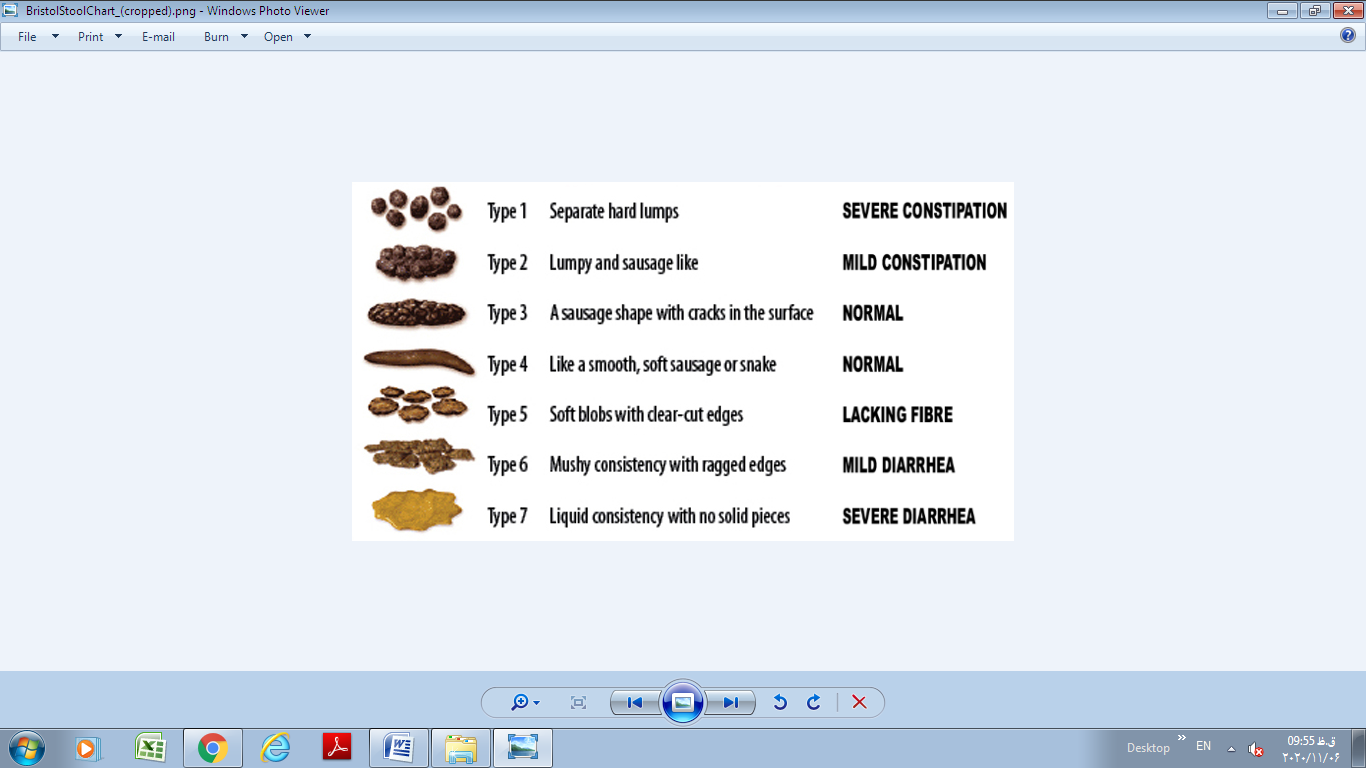


**Fourth part: Colonoscopy finding**

Skin tag 🞎 Pinworm 🞎 Erosion 🞎 Small Sessile polyp 🞎 NonSmall Sessile polyp 🞎 Internal Hemorrhoid🞎 Diverticulum 🞎 ulcer🞎 mass 🞎 Adenomatous polyp 🞎

Vascularity 🞎 Adenocarcinoma 🞎 Nodularity 🞎

**Location of the lesion**

Sigmoid Colon🞎 Descending Colon🞎 Transverse Colon 🞎 Ascending Colon🞎 Cecum 🞎 rectum🞎 Anus and anus canal🞎

Having Colon Polyp: No 🞎 Yes🞎

Polyp Number: 1🞎 2 🞎 3🞎

Polyp size: At least one small polyp 🞎 At least one large polyp🞎
