# Supplementary material for: The frequency of colorectal lesions in the first-degree relatives of patients with colorectal lesions among PERSIAN Guilan Cohort Study population (PGCS)
Source: BMC Gastroenterol. 2024 Feb 26;24:88. doi: 10.1186/s12876-024-03177-z (PMC10898130; doi:10.1186/s12876-024-03177-z)
Supplement: Supplementary file 2 — Supplementary Material 2 [file 12876_2024_3177_MOESM2_ESM.doc]

STROBE Statement—checklist of items that should be included in reports of observational studies

|  | Item No | Recommendation |
| --- | --- | --- |
| **Title and abstract** | 1 | (*a*) Indicate the study’s design with a commonly used term in the title or the abstract✓  Title:The frequency of colon lesions in the first-degree relatives of patients with adenomatous polyp or premalignant/ malignant colon lesions referring to the PERSIAN Guilan Cohort Study (PGCS).  Abstract:In this cross-sectional study in the prospective epidemiological research studies of Iranian adults (PERSIAN) Guilan Cohort Study (PGCS) population |
|  |  | (*b*) Provide in the abstract an informative and balanced summary of what was done and what was found ✓  Considering the high prevalence of neoplastic colon polyps among the first-degree relatives of the patients with adenomatous polyp, early screening is recommended for the people with family history of this disease in the same way that people with family history of colorectal cancer (CRC) are screened |
| Introduction | | |
| Background/rationale | 2 | Explain the scientific background and rationale for the investigation being reported✓  However, the British Society of Gastroenterology recommends screening only for people with a family history of colorectal cancer and disregards those with a family history of polyp . To shed light on this issue, the present study aimed to investigate the frequency of colon lesions among the first-degree relatives of the patients with adenomatous polyp or premalignant/ malignant colon lesions referring to the geriatric cohort of Guilan |
| Objectives | 3 | State specific objectives, including any prespecified hypotheses✓  The aim of this study was to investigate the frequency of colonic lesions in the first-degree relatives of patients with adenomatous polyp or premalignant/ malignant colon lesions referring to the geriatric cohort of Guilan |
| Methods | | |
| Study design | 4 | Present key elements of study design early in the paper✓  In this cross-sectional study in the prospective epidemiological research studies of Iranian adults (PERSIAN) Guilan Cohort Study (PGCS) population, 162 subjects who were the first-degree relatives of 52 participants over 50 years old in PGCS with adenomatous polyp were investigated and underwent total colonoscopy based on the guidelines proposed for screening colorectal cancer |
| Setting | 5 | Describe the setting, locations, and relevant dates, including periods of recruitment, exposure, follow-up, and data collection✓  In this cross-sectional study from November 2021 to May 2022, the first-degree relatives (brothers, sisters, sons, and daughters) of the patients with adenomatous polyp, who were diagnosed with the disease in the course of another research project. These patients were people aged above 50 years who participated in the PERSIAN Guilan Cohort Study (PGCS) as a part of the PERSIAN (Prospective Epidemiological Research Studies in IRAN) cohort [15,16] with a sample size of 10,520 males and females in Some’e Sara County (including 39 villages and urban regions), Guilan, Iran [17]. The average household size in Guilan Province has been reported 3.13 in the Statistical Information System of the province. Accordingly, we decided to investigate 3 people from each of the families that had a patient with polyp |
| Participants | 6 | (*a*) *Cohort study*—Give the eligibility criteria, and the sources and methods of selection of participants. Describe methods of follow-up  *Case-control study*—Give the eligibility criteria, and the sources and methods of case ascertainment and control selection. Give the rationale for the choice of cases and controls  *Cross-sectional study*—Give the eligibility criteria, and the sources and methods of selection of participants🞎  162 subjects who were the first-degree relatives of 52 participants over 50 years old in PGCS with adenomatous polyp were investigated and underwent total colonoscopy based on the guidelines proposed for screening colorectal cancer |
|  |  | (*b*) *Cohort study*—For matched studies, give matching criteria and number of exposed and unexposed  *Case-control study*—For matched studies, give matching criteria and the number of controls per case |
| Variables | 7 | Clearly define all outcomes, exposures, predictors, potential confounders, and effect modifiers. Give diagnostic criteria, if applicable✓  Moreover, the subjects’ demographic information, colonoscopy results, and pathological findings were recorded using a checklist Logistic regression with backward elimination (p-value for removal >0.1) was used to identify factors associated with presence of colon polyps. Odds ratio (OR) and 95% confidence interval (CI) were calculated |
| Data sources/ measurement | 8* | For each variable of interest, give sources of data and details of methods of assessment (measurement). Describe comparability of assessment methods if there is more than one group✓  In this study, continuous variables were expressed as mean ± standard deviation (SD) and categorical variables as number (percentage). In univariable analysis, simple logistic regression was used to examine the relationship of demographic and clinical variables with the presence of colon polyps. Then, backward logistic regression analysis was applied to identify the independent risk factors for the outcome. In this analysis, the alpha-to-remove was set at 0.1. Odds ratio (OR) and 95% confidence interval (CI) were calculated. All data analyses were done with SPSS for Windows, version 16.0 (SPSS Inc., Chicago, IL, USA), and level of significance was set at 0.05 |
| Bias | 9 | Describe any efforts to address potential sources of bias🞎(Noun) |
| Study size | 10 | Explain how the study size was arrived at✓  Based on the obtained information, the sample size of 150 was deemed appropriate for our purpose. Finally, 162 subjects entered the study. For the purpose of the study, the list of the patients with adenomatous polyp or colorectal cancer was first obtained from PGCS. Then, the researchers contacted each of them and asked them to give the characteristics of their first-degree relatives. After that, the first-degree relatives were contacted randomly, and the purpose of the study was fully explained to them |
| Quantitative variables | 11 | Explain how quantitative variables were handled in the analyses. If applicable, describe which groupings were chosen and why✓  In univariable analysis, simple logistic regression was used to examine the relationship of demographic and clinical variables with the presence of colon polyps. Then, backward logistic regression analysis was applied to identify the independent risk factors for the outcome. In this analysis, the alpha-to-remove was set at 0.1. Odds ratio (OR) and 95% confidence interval (CI) were calculated. All data analyses were done with SPSS for Windows, version 16.0 (SPSS Inc., Chicago, IL, USA), and level of significance was set at 0.05 |
| Statistical methods | 12 | (*a*) Describe all statistical methods, including those used to control for confounding🞎(Noun) |
|  |  | (*b*) Describe any methods used to examine subgroups and interactions✓  In this study, continuous variables were expressed as mean ± standard deviation (SD) and categorical variables as number (percentage). In univariable analysis, simple logistic regression was used to examine the relationship of demographic and clinical variables with the presence of colon polyps. Then, backward logistic regression analysis was applied to identify the independent risk factors for the outcome. In this analysis, the alpha-to-remove was set at 0.1. Odds ratio (OR) and 95% confidence interval (CI) were calculated. All data analyses were done with SPSS for Windows, version 16.0 (SPSS Inc., Chicago, IL, USA), and level of significance was set at 0.05 |
|  |  | (*c*) Explain how missing data were addressed🞎(Noun) |
|  |  | (*d*) *Cohort study*—If applicable, explain how loss to follow-up was addressed  *Case-control study*—If applicable, explain how matching of cases and controls was addressed  *Cross-sectional study*—If applicable, describe analytical methods taking account of sampling strategy🞎(Noun) |
|  |  | (*e*) Describe any sensitivity analyses |

Continued on next page

| Results | | |
| --- | --- | --- |
| Participants | 13* | (a) Report numbers of individuals at each stage of study—eg numbers potentially eligible, examined for eligibility, confirmed eligible, included in the study, completing follow-up, and analysed✓  Based on the obtained information, the sample size of 150 was deemed appropriate for our purpose. Finally, 162 subjects entered the study. For the purpose of the study, the list of the patients with adenomatous polyp or colorectal cancer was first obtained from PGCS. Then, the researchers contacted each of them and asked them to give the characteristics of their first-degree relatives. After that, the first-degree relatives were contacted randomly, and the purpose of the study was fully explained to them |
|  |  | (b) Give reasons for non-participation at each stage🞎(Noun) |
|  |  | (c) Consider use of a flow diagram🞎(Noun) |
| Descriptive data | 14* | (a) Give characteristics of study participants (eg demographic, clinical, social) and information on exposures and potential confounders✓  Based on the obtained information, the sample size of 150 was deemed appropriate for our purpose. Finally, 162 subjects entered the study. For the purpose of the study, the list of the patients with adenomatous polyp or colorectal cancer was first obtained from PGCS. Then, the researchers contacted each of them and asked them to give the characteristics of their first-degree relatives. After that, the first-degree relatives were contacted randomly, and the purpose of the study was fully explained to them |
|  |  | (b) Indicate number of participants with missing data for each variable of interest🞎(Noun) |
|  |  | (c) *Cohort study*—Summarise follow-up time (eg, average and total amount) 🞎 |
| Outcome data | 15* | *Cohort study*—Report numbers of outcome events or summary measures over time |
|  |  | *Case-control study—*Report numbers in each exposure category, or summary measures of exposure |
|  |  | *Cross-sectional study—*Report numbers of outcome events or summary measures✓  In total, 86 colon polyps (52 neoplastic and 34 non-neoplastic polyps) were observed in 56 (34.6%) individuals. Being older, higher BMI, cigarette smoking, and diabetes were significantly associated with a greater risk of having colon polyps. In contrast, higher consumption of vegetables as well as fruits were significantly associated with a less risk of having colon polyps |
| Main results | 16 | (*a*) Give unadjusted estimates and, if applicable, confounder-adjusted estimates and their precision (eg, 95% confidence interval). Make clear which confounders were adjusted for and why they were included🞎(Noun) |
|  |  | (*b*) Report category boundaries when continuous variables were categorized✓  Age group  40-50 y  50-60 y  >60 y |
|  |  | (*c*) If relevant, consider translating estimates of relative risk into absolute risk for a meaningful time period🞎(Noun) |
| Other analyses | 17 | Report other analyses done—eg analyses of subgroups and interactions, and sensitivity analyses🞎(Noun) |
| Discussion | | |
| Key results | 18 | Summarise key results with reference to study objectives✓  Being older, higher BMI, cigarette smoking, and diabetes were significantly associated with a greater risk of having colon polyps. In contrast, higher consumption of vegetables as well as fruits were significantly associated with a less risk of having colon polyps.  Considering the high prevalence of neoplastic colon polyps among the first-degree relatives of the patients with adenomatous polyp, early screening is recommended for the people with family history of this disease in the same way that people with family history of colorectal cancer (CRC) are screened |
| Limitations | 19 | Discuss limitations of the study, taking into account sources of potential bias or imprecision. Discuss both direction and magnitude of any potential bias✓  The present study has several limitations that should be considered when interpreting the findings. First, the sample size was relatively small, which may have reduced the statistical power to detect factors associated with the presence of polyp colon. Second, the particular conditions of the province in terms of the COVID-19 pandemic, which limited our access to more participants, and the lack of a control group were limitations of the study. |
| Interpretation | 20 | Give a cautious overall interpretation of results considering objectives, limitations, multiplicity of analyses, results from similar studies, and other relevant evidence✓  Based on the findings obtained in this study and regarding the high prevalence of neoplastic colon polyps among the first-degree relatives of patients with adenomatous polyp, it seems reasonable to carry out early screening for people with a family history of such polyps in the same way that people with a family history of colorectal cancer are screened |
| Generalisability | 21 | Discuss the generalisability (external validity) of the study results🞎(Noun) |
| Other information | | |
| Funding | 22 | Give the source of funding and the role of the funders for the present study and, if applicable, for the original study on which the present article is based✓  We thank all subjects for participating in this study and the Guilan University of Medical Science, Rasht, Iran for supporting this study |

*Give information separately for cases and controls in case-control studies and, if applicable, for exposed and unexposed groups in cohort and cross-sectional studies.

**Note:** An Explanation and Elaboration article discusses each checklist item and gives methodological background and published examples of transparent reporting. The STROBE checklist is best used in conjunction with this article (freely available on the Web sites of PLoS Medicine at http://www.plosmedicine.org/, Annals of Internal Medicine at http://www.annals.org/, and Epidemiology at http://www.epidem.com/). Information on the STROBE Initiative is available at www.strobe-statement.org.
